# Extracardiac Manifestations Fail to Predict the Severity of Cardiac Phenotype in Children and Young Adults with Marfan Syndrome

**DOI:** 10.21203/rs.3.rs-3994693/v1

**Published:** 2024-03-04

**Authors:** Sheba John, Luciana T. Young, Ronald V. Lacro, Arvind Hoskoppal, Zhining Ou, Angela Presson, Joyce T. Johnson, Lauren Andrade, L. LuAnn Minich, Shaji Menon

**Affiliations:** University of Utah School of Medicine; University of Washington School of Medicine; Harvard Medical School; University of Pittsburgh School of Medicine; University of Utah School of Medicine; University of Utah School of Medicine; John Hopkins All Children’s Hospital; Maine Medical Center; University of Utah School of Medicine; UPMC Heart and Vascular Institute

**Keywords:** Marfan syndrome, extracardiac manifestations, cardiac phenotype

## Abstract

We performed a secondary analysis of the Pediatric Heart Network Marfan Trial public-use database to evaluate associations between extracardiac features and cardiac and aortic phenotypes in study participants. Aortic aneurysm phenotype was defined as aortic root Z-score ≥ 4.5, aortic root growth rate ≥ 75th percentile, aortic dissection, and aortic surgery. Severe cardiac phenotype was defined as aortic dissection, aortic Z-score ≥4.5, aortic valve surgery, at least moderate mitral regurgitation, mitral valve surgery, left ventricular dysfunction, or death. Extracardiac manifestations were characterized by specific organ system involvement and by a novel aggregate extracardiac score that was created for this study based on the original Ghent nosology. Logistic regression analysis compared aggregate extracardiac score and systems involvement to outcomes. Of 608 participants (60% male), the median age at enrollment was 10.8 years (interquartile range: 6, 15.4). Aortic aneurysm phenotype was observed in 71% of participants and 64% had severe cardiac phenotype. On univariate analysis, skeletal (OR: 1.95, 95% CI: 1.01, 3.72; p = 0.05), skin manifestation (OR: 1.62, 95% CI: 1.13, 2.34; p = 0.01) and aggregate extracardiac score (OR: 1.17, 95% CI: 1.02, 1.34; p = 0.02) were associated with aortic aneurysm phenotype but were not significant in multivariate analysis. There was no association between extracardiac manifestations and severe cardiac phenotype. Thus, the severity of cardiac manifestations in Marfan syndrome was independent of extracardiac phenotype and aggregate extracardiac score. Severity of extracardiac involvement did not appear to be a useful clinical marker for cardiovascular risk-stratification in this cohort of children and young adults with Marfan syndrome.

## Introduction

Marfan syndrome (MFS) is a heritable systemic connective tissue disorder occurring in 1 in 5,000 individuals. Caused by mutations in the *FBN1* gene, MFS has multiorgan involvement affecting primarily the ocular, musculoskeletal and cardiovascular systems (1–4). Cardiovascular manifestations, including progressive aortic enlargement, aortic dissection and rupture, mitral regurgitation requiring surgical intervention, and left ventricular dysfunction, are associated with significant morbidity and mortality (5,6). Risk for aortic dissection and aortic surgery increases with increasing aortic root diameter, and thresholds for medical and surgical therapy are largely dependent on aortic root size. However, aortic root size is not a perfect predictor. For example, prophylactic aortic root replacement is generally recommended at an aortic root diameter of 5 cm, yet 15% of cases of aortic dissection in Marfan syndrome occur at aortic diameters < 5 cm (7,8). Although cardiac and extracardiac phenotypes have been well-characterized, the relationship between extracardiac manifestations and cardiovascular outcomes remain unclear. If extracardiac phenotypes positively correlate with the severity of cardiovascular involvement, the association may be useful for risk-stratification and clinical management. Therefore, the primary objective of this study was to evaluate the association between extracardiac features and severe aortic and cardiac phenotypes in children, adolescents, and young adults with MFS utilizing the large multicenter Pediatric Heart Network Marfan (PHN) Trial public-use dataset.

## Methods

### Study design:

We performed a secondary analysis of the PHN Marfan Trial public-use database. The PHN Marfan Trial was a randomized clinical trial comparing the effects of beta blocker (atenolol) therapy versus angiotensin II receptor blocker (losartan) therapy on the rate of aortic root enlargement in children and young adults with MFS. A detailed description of trial design and results has previously been reported (9,10). Briefly, between January 2007 and February 2011, 608 participants, aged 6 months to 25 years, diagnosed with MFS using the original Ghent criteria (11) who had an aortic root Z-score >3 and aortic root diameter <5 cm were enrolled in the trial. Individuals with previous aortic surgery, planned aortic surgery within 6 months of enrollment, or history of aortic dissection were excluded. The study enrollment forms collected detailed data regarding extracardiac features, organ system involvement, and Ghent nosology criteria for all participants at five time periods (baseline, 6 months, 12 months, 24 months and 36 months). The protocol was approved by the Institutional Review or Ethics Board at each participating site.

### Outcomes and definitions:

Primary outcomes for this analysis included aortic aneurysm phenotype and severe cardiac phenotype.

- **Aortic aneurysm phenotype** was defined as having an aortic root Z-score ^3^4.5, aortic annual growth rate of ^3^75^th^ percentile based on change in aortic root Z-score during the trial, aortic surgery, or aortic dissection.
- **Severe cardiac phenotype** was defined as aortic dissection, aortic Z-score ^3^4.5, aortic valve surgery, at least moderate mitral regurgitation, mitral valve surgery at the time of trial enrollment, left ventricular dysfunction (ejection fraction £45%), or death.
- **Extracardiac systems** included musculoskeletal, ocular, dural, pulmonary, and skin manifestations. Based on the original Ghent nosology, a novel **aggregate extracardiac score** (AES) was created for this study to evaluate multiorgan involvement. Extracardiac manifestations meeting **major**criteria were assigned two points and those findings meeting **minor** criteria were assigned one point. **AES** was derived by adding the number of qualifying minor and major criteria for each participant (Table 1).

**Table:** 

*Extracardiac Manifestations by System with Major and Minor Criteria*			
Systems		*Major*	*Minor*
*Skeletal*	a. Pectus carinatum	Presence of ≥4 items among (a)-(h)	Presence of 2–3 items among (a) – (h) OR presence of 1 item among (a)-(h) and presence 2 items from (i)-(l)
	b. Pectus excavatum - moderate to severe		
	c. Reduced upper-to-lower segment ratio for age OR arm span-to-height ratio > 1.05		
	d. Wrist AND thumb signs		
	e. Scoliosis > 20° OR spondylolisthesis		
	f. Reduced extension at the elbow (< 170°)		
	g. Pes planus		
	h. Protrusio acetabuli of any degree		
	i. Mild pectus excavatum		
	j. Joint hypermobility		
	k. Highly arched palate		
	l. Facial features		
*Ocular*	a.Ectopia lentis	Presence of (a)	Presence of one item from (b)-(d)
	b. Flat cornea		
	c. Increased axial length of the globe		
	d. Hypoplastic iris OR hypoplastic ciliary muscle causing decreased miosis		
*Pulmonary*	a. Spontaneous pneumothorax		Presence of one item from (a)-(b)
	b. Apical blebs (by chest X-ray, CT or MRI)		
*Skin and Integument*	a. Striae distensae		Presence of one item from (a)-(b)
	b. Recurrent or incisional hernia		
*Dural*	a. Lumbosacral dural ectasia (by CT or MRI)	Presence of (a)

### Statistical analysis:

Demographics and clinical outcomes of interest were summarized using median and interquartile range (IQR) for continuous variables; counts and percentages were used for categorical variables. Univariate mixed effect logistic regression models were created, accounting for center-to-center variabilities, to evaluate the association between risk factors and outcomes (aortic aneurysm phenotype and severe cardiac phenotype). Multivariate mixed effects logistic regressions included risk factors that were significant at level 0.10 from univariate analysis including baseline aortic root elastic modulus, and baseline aortic root stiffness index for severe cardiac phenotype outcome. For aortic aneurysm phenotype, the multivariable model included baseline age at echocardiography, family and genetic history of MFS, family history of aortic dissection, treatment (losartan, atenolol), baseline aortic root elastic modulus, baseline aortic root stiffness index, skeletal, skin manifestation, aggregate extracardiac score (AES). Odds ratio and 95% confidence interval (CI) were reported. Statistical significance was assessed at the 0.05 level, unless specified otherwise as above for variable selection. Statistical analyses were implemented using R v. 4.1.2 (13).

## Results

### Participant characteristics:

All 608 participants in the PHN Marfan trial who met the original diagnostic Ghent criteria were included in this secondary analysis. Median age at enrollment was 10.8 years (IQR 6 to15.4 years) and 366 participants (60%) were males.

### Cardiovascular phenotypes:

**Figure 1:**
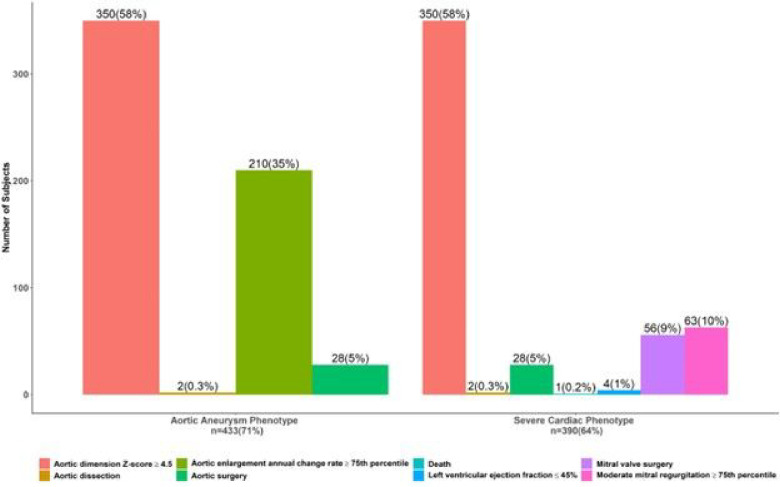
Cardiovascular Outcomes Among PHN Marfan Trial Participants

The prevalence of cardiovascular outcomes among PHN Marfan trial participants are summarized in Figure 1.

Of the 608 participants, 433 (71%) met criteria for the aortic aneurysm phenotype: 350 (58%) had an aortic root Z-score ^3^4.5, 210 (35%) had an aortic root growth rate ≥75^th^ percentile, 28 (5%) underwent aortic surgery, and only 2 (0.3%) had an aortic dissection. Of the 608 participants, 390 (64%) met the criteria for the severe cardiac phenotype: 63 (10%) had at least moderate mitral regurgitation, 56 (9%) underwent mitral valve surgery, 4 (1%) had left ventricular dysfunction (ejection fraction £45%). There was 1 death in the PHN Marfan Trial related to heart failure. Overall, a total of 81 (13%) participants had major cardiac complications including aortic valve surgery, mitral valve surgery, aortic dissection, or death.

### Extracardiac Phenotype:

The prevalence of major and minor criteria for each extracardiac system and the aggregate extracardiac score are shown in Table 2.

**Table:** 

*a. Extracardiac Manifestations*		
	*N=608*	
*Systems*	*Categories*	*N*
*Skeletal*	Major	289
	Minor	279
*Ocular* ^	Major	252
	Minor	27
*Pulmonary*	Minor	18
*Skin and Integument*	Minor	266
*Dural* *	Major	29
*b. Aggregate Extracardiac Score*		
	*N =608*	
*Points*	*N (%)*	
*1*	106 (17.4)	
*2*	102 (16.8)	
*3*	196 (32.2)	
*4*	116 (19.1)	
*5*	58 (9.5)	
*6*	7 (1.2)	
*7*	1 (0.2)	

^ 520 participants with ocular data

* 92 participants screened for dural ectasia

The skeletal and ocular systems had both major and minor criteria, dural had only major criteria, and pulmonary and skin manifestations had only minor criteria. Of the 608 participants, 289 (48%) met major skeletal criteria, 18 (3%) had at least one pulmonary manifestation (apical blebs or spontaneous pneumothorax), and 266 (44%) had at least one skin (striae or hernia) manifestation. Among the 520 participants with available ocular data, 252 met major ocular criteria (ectopia lentis). MRI data was available for 92 participants of which 32 had lumbosacral dural ectasia. Pulmonary involvement had the lowest prevalence among all extracardiac systems included in Marfan Syndrome criteria. Only 11% of participants had severe extracardiac involvement as indicated by an aggregate extracardiac score ≥5.

### Associations of extracardiac manifestations, aggregate extracardiac score, and cardiovascular involvement:

On univariate analysis, skeletal, skin manifestations, and aggregate extracardiac score were associated with aortic aneurysm phenotype (OR: 1.95, 95% CI: 1.01, 3.72; p=0.05; OR: 1.62, 95% CI: 1.13, 2.34; p=0.01; and OR: 1.17, 95% CI: 1.02, 1.34; p=0.02 respectively, Table 3). However, no independent association was found on multivariate analysis. There was no association between extracardiac manifestations or aggregate extracardiac score with severe cardiac phenotype.

**Table:** 

	Severe cardiac phenotype		Aortic aneurysm phenotype	
E*xtracardiac Manifestations*	*OR (95% CI)*	*p-value*	*OR (95% CI)*	*p-value*
*Skeletal*	1.34 (0.7, 2.55)	0.37	1.95 (1.01, 3.72)	0.05
*Ocular*	1.09 (0.76, 1.57)	0.63	1.01 (0.69, 1.48)	0.97
*Pulmonary*	2.59 (0.89, 10.03)	0.08	0.77 (0.3, 2.15)	0.59
*Skin*	1.01 (0.72, 1.41)	0.97	1.62 (1.13, 2.34)	0.01
*Dural*	1.53 (0.58, 4.3)	0.39	1.02 (0.39, 2.77)	0.97
*Aggregate Extracardiac Score*	1.10 (0.96, 1.25)	0.17	1.17 (1.02, 1.34)	0.02

## Discussion

In Marfan syndrome, the risk for aortic dissection and aortic surgery increases with increasing aortic root diameter. Unfortunately, absolute aortic root size is not a great predictor for aortic dissection and/or rupture (7,8). Our analysis used the data from one of the first large multicenter studies of a well characterized MFS cohort to evaluate the relationship between extracardiac manifestations and aggregate extracardiac score and severity of cardiovascular system involvement. Our key finding was absence of an association between specific organ system involvement or aggregate extracardiac score and cardiovascular outcomes in MFS. Although skeletal manifestations, skin manifestations and aggregate extracardiac score were associated with aortic aneurysm phenotype in univariate analysis, these associations lost significance in multivariate models.

Accurate risk-stratification and timely interventions can save lives but evaluating the risk of aortic dissection or rupture in the population with MFS remains challenging. Although it is compelling to think that the severity of the more visible manifestations of MFS may correlate with the severity of the cardiac findings, few studies have assessed the association of extracardiac findings with cardiovascular phenotype (15). Previously reported risk factors for aortic dissection and severe cardiac phenotype include prior prophylactic aortic surgery, aortic diameter, and pregnancy (16). An increased prevalence, but not severity, of mitral valve prolapse was reported in individuals with MFS and kyphoscoliosis (17). The Hungarian Marfan Registry (HMR) study found aortic dissection was more common in participants with striae atrophicae. Although we found an association between skin manifestation and severe aortic phenotype on univariate analysis, this association lost its significance in multivariate modeling. One explanation for this discrepancy may be the older age of HMR participants (33.2 ±13.5 years) compared to the PHN Marfan trial participants (median age 10.8 years at enrollment). The weighting of major and minor criteria for calculating the aggregate extracardiac score was somewhat arbitrary, based on the original Ghent criteria. It is possible that a different weighting system might uncover different associations. Regardless, the aggregate extracardiac score did not independently predict a severe aortic or cardiac phenotype, as defined in our study.

## Limitations

The entry criteria for the PHN Marfan Trial excluded children and young adults with Marfan syndrome at the mild (aortic root z-score < 3) and severe (those who already had aortic surgery or aortic dissection) ends of the spectrum. Although this cohort was well characterized both for extracardiac and cardiac phenotypes, the follow-up period was only 3 years. It is possible that associations between extracardiac phenotype and cardiovascular outcomes such as aortic surgery and aortic dissection might be found after a longer period of follow-up. Aggregate extracardiac score derived from the original Ghent nosology used in this study has not been previously validated. Though the large multicenter PHN Marfan Trial public-use database provides a large patient cohort, the phenotypic features of the original Ghent nosology have since been revised to put more emphasis on aortic root dilation and ectopia lentis, with findings in other organ systems contributing to a systemic score (14). The systemic score was not evaluated in this study. In addition, we had no molecular data and could not explore the prior reports of an association of elevated circulating TGF-ß levels or increased expression of MMP-3 with aortic dissection (18).

## Conclusion

Despite great advances in care, cardiovascular risk stratification in MFS remains challenging. Our secondary analysis of the PHN Marfan Trial public-use database showed that extracardiac phenotype failed to predict the severity of cardiovascular involvement (as defined by our study), and thus, has limited value for risk-stratification or management. Therefore, all individuals with MFS, regardless of their extracardiac phenotype, should be followed closely with serial surveillance imaging throughout their lifetime. Aortic root size, while not perfect, remains the best predictor of major aortic complications in MFS. Future studies may focus on the association between the revised Ghent systemic score, molecular and genetic data, and severity of cardiovascular manifestations to better risk stratify MFS patients for cardiac complications.

## Highlights

- We performed a secondary analysis of the Pediatric Heart Network Marfan Trial public-use database to evaluate associations between extracardiac features and cardiac and aortic phenotypes.
- Although skeletal and skin manifestations and aggregate extracardiac score were associated with aortic aneurysm phenotype in univariate analysis, they were not significant in multivariate models.
- Severity of cardiovascular disease in MFS was independent of extracardiac manifestations or aggregate extracardiac score.

## References

[1] Judge DP, Dietz HC. Marfan’s syndrome. Lancet 2005;366:1965–1976. 16325700 10.1016/S0140-6736(05)67789-6 PMC1513064

[2] Silverman DI, Burton KJ, Gray J, Bosner MS, Douchoukos NT, Roman MJ, Boxer M, Devereux RB, Tsipoura P. Life expectancy in Mafan syndrome. Am J Cardiol 1995;75(2):157–160. 7810492 10.1016/s0002-9149(00)80066-1

[3] Baer RW, Taussig HB, Oppenheimer EH. Congenital aneurysmal dilatation of the aorta associated with arachnodactyly. Bull Johns Hopkins Hosp 1943;72:309–331.

[4] Etter LE, Glover LP. Arachnodactyly complicated by dislocated lens and death from rupture of the dissecting aneurysm JAMA 1943;123:88–89.

[5] Hiratzka LF, Bakris GL, Beckman JA, 2010 ACCF/AHA/AATS/ACR/ASA/SCA/SCAI/SIR/STS/SVM guidelines for the diagnosis and management of patients with Thoracic Aortic Disease: a report of the American College of Cardiology Foundation/American Heart Association Task Force on Practice Guidelines, American Association for Thoracic Surgery, American College of Radiology, American Stroke Association, Society of Cardiovascular Anesthesiologists, Society for Cardiovascular Angiography and Interventions, Society of Interventional Radiology, Society of Thoracic Surgeons, and Society for Vascular Medicine [published correction appears in Circulation. 2010 Jul 27;122(4):e410]. Circulation. 2010;121(13):e266–e369. 20233780 10.1161/CIR.0b013e3181d4739e

[6] Knadler JJ, LeMaire S, McKenzie ED, Moffett B, Morris SA. Thoracic Aortic, Aortic Valve, and Mitral Valve Surgery in Pediatric and Young Adult Patients With Marfan Syndrome: Characteristics and Outcomes. Semin Thorac Cardiovasc Surg. 2019;31(4):818–825. doi:10.1053/j.semtcvs.2019.06.005 31233783 PMC7473414

[7] Svensson LG, Khitin L. Aortic cross-sectional area/height ratio timing of aortic surgery in asymptomatic patients with Marfan syndrome. J Thorac Cardiovasc Surg. 2002;123(2):360–361. doi:10.1067/mtc.2002.118497 11828302

[8] Gott VL, Greene PS, Alejo DE, Replacement of the aortic root in patients with Marfan’s syndrome. N Engl J Med. 1999;340(17):1307–1313. doi:10.1056/NEJM199904293401702 10219065

[9] Lacro RV, Dietz HC, Wruck LM, Bradley TJ, Colan SD, Devereux RB, Klein GL, Li JS, Minich LL, Paridon SM, Pearson GD, Printz BF, Pyeritz RE, Radojewski E, Roman JM, Saul JP, Stylianou MP, Mahony L, for the Pediatric Heart Network Investigators. Rationale and design of a randomized clinical trial of beta-blocker therapy (atenolol) versus angiotensin II receptor blocker therapy (losartan) in individuals with Marfan syndrome. Am Heart J 2007;154:624–631. 17892982 10.1016/j.ahj.2007.06.024 PMC3042860

[10] Lacro RV, Dietz HC, Sleeper LA, Yetman AT, Bradley TJ, Colan SD, Pearson GD, Selamet Tierney ES, Levine JC, Atz AM, Benson DW, Braverman AC, Chen S, De Backer J, Gelb BD, Grossfeld PD, Klein GL, Lai WW, Liou A, Loeys BL, Markham LW, Olson AK, Paridon SM, Pemberton VL, Pierpont ME, Pyeritz RE, Radojewski E, Roman MJ, Sharkey AM, Stylianou MP, Wechsler SB, Young LT, Mahony L, Pediatric Heart Network Investigators. Atenolol versus losartan in children and young adults with Marfan’s syndrome. N Engl J Med 2014; 371(22):2061–2071. 25405392 10.1056/NEJMoa1404731 PMC4386623

[11] De Paepe A, Devereux RB, Dietz HC, Hennekam RC, Pyeritz RE. Revised diagnosis criteria for the Marfan syndrome. Am J Med Genet 1996;62:417–426. 8723076 10.1002/(SICI)1096-8628(19960424)62:4<417::AID-AJMG15>3.0.CO;2-R

[12] De Backer J. Cardiovascular characteristics in Marfan syndrome and their relation to the genotype. Verh K Acad Geneeskd Belg 2009;71(6) 2009;71(6):335–371. 20232788

[13] Team, R. Core. “R: A language and environment for statistical computing.” (2013): 201.

[14] Loeys BL, Dietz HC, Braverman AC, Callewaert BL, De Backer J, Devereux RB, Hillhorst-Hofstee Y, Jondeau G, Faivre L, Milewicz DM, Pyeritz RE, Sponseller PD, Wordsworth P, De Paepe AM. The revised Ghent nosology for the Marfan syndrome. J Med Genet 2010;47:476–485. 20591885 10.1136/jmg.2009.072785

[15] Faivre L, Collod-Beroud G, Loeys BL, Child A, Binquet C, Gautier E, Callewaert B, Arbustini E, Mayer K, Arslan-Kirchner M, Kiotsekoglou A, Comeglio P, Marziliano N, Dietz HC, Halliday D, Beroud C, Bonithon-Kopp C, Claustres M, Muti C, Plauchu H, Robinson PN, Adès LC, Biggin A, Benetts B, Brett M, Holman KJ, De Backer J, Coucke P, Francke U, De Paepe A, Jondeau G, Boileau C. Effect of mutation type and location on clinical outcome in 1,013 probands with Marfan syndrome or related phenotypes and FBN1 mutations: an international study. J Hum Genet 2007 Sep;81(3):454–466. 10.1086/520125 PMC1950837 17701892

[16] den Hartog AW, Franken R, Zwinderman AH, The risk for type B aortic dissection in Marfan syndrome. J Am Coll Cardiol. 2015;65(3):246–254. doi:10.1016/j.jacc.2014.10.050 25614422

[17] Bruno L, Tredici S, Mangiavacchi M, Colombo V, Mazzotta GF, Sirtori CR. Cardiac, skeletal, and ocular abnormalities in patients with Marfan’s syndrome and in their relatives. Comparison with the cardiac abnormalities in patients with kyphoscoliosis. Br Heart J 1984;51(2):220–230. 6691872 10.1136/hrt.51.2.220 PMC481487

[18] Ágg B, Benke K, Szilveszter B, Polós M, Daróczi L, Odler B, Nagy AB, Tarr F, Merkely B, Szabolcs Z. Possible extracardiac predictors of aortic dissection in Marfan syndrome. BMC Cardiovascular Disorders 2014;14:47. 24720641 10.1186/1471-2261-14-47 PMC4021409

